# Supplementary material for: Specific Non-Reducing Ends in Heparins from Different Animal Origins: Building Blocks Analysis Using Reductive Amination Tagging by Sulfanilic Acid
Source: Molecules. 2020 Nov 26;25(23):5553. doi: 10.3390/molecules25235553 (PMC7730200; doi:10.3390/molecules25235553)
Supplement: Supplementary file 1 [file molecules-25-05553-s001.pdf]

Supplementary data

Specific non-reducing ends in heparins from different animal origins: building blocks analysis using reductive amination tagging by sulfanilic acid

Pierre Mourier*, Sanofi, Vitry-sur-Seine, France

Table of contents	Page
**Isolation and identification of NRE tetrasaccharides in semuloparin fraction**	S-3
Figure S1. Chromatogram on AS11 (accelerated gradient) of the of sulfanilic tagged Semuloparin tetrasaccharide fraction (— Signal specific of saturated tetrasaccharides)	S-3
Figure S2. Semi preparative chromatogram on AS11 (column 250x21mm) of the of sulfanilic tagged Semuloparin tetrasaccharide fraction (— Signal specific of saturated tetrasaccharides)	S-4
Figure S3: Chromatogram on AS11 of the 2 purified sulfanilic tagged NRE tetrasaccharides (— Signal specific of saturated tetrasaccharides)	S-4
NMR characterization	S-5
Table S1: Proton and carbon chemical shifts for GlcA^{IV}-Glc2NS3S6S^{III}-IdoA2S^{II}-Glc2NS6S^{I} (<u>IIs$_{glu}$</u>-Is$_{id}$)^{sulf}	S-5
Figure S4: Structure of the tetrasaccharide <u>IIs$_{glu}$</u>-Is$_{id}$ after reductive amination by sulfanilic acid	S-5
Figure S5: ^{1}H spectrum of <u>IIs$_{glu}$</u>-Is$_{id}^{sulf}$ (D2O, 25°C, 500 MHz)	S-6
Figure S6: Structure of the minor NRE tetrasaccharide Is$_{id}$-Is$_{id}$ after reductive amination by sulfanilic acid.	S-6
Table S2: Proton and carbon chemical shifts for IdoA2S^{IV}-Glc2NS6S^{III}-IdoA2S^{II}-Glc2NS6S^{I} (Is$_{id}$-Is$_{id}$)^{sulf}	S-7
Figure S7: ^{1}H spectrum of Is$_{id}$Is$_{id}^{sulf}$ (D2O, 25°C, 500 MHz)	S-7
**Isolation and identification of NRE disaccharides in disaccharide fractions from digested heparins**	S-8
Figure S8: Chromatogram on AS11 (accelerated gradient) of the purified NRE building blocks Is$_{id}^{sulf}$ (b) and GlcNS,6S^{sulf} (c) from sulfanilic tagged disaccharide fraction of bovine lung heparin digest (heparinase 1) (a)	S-8
Figure S9: Semi preparative chromatogram on AS11 (column 250x21mm) of the sulfanilic tagged bovine lung heparin digest (hep I) disaccharide fraction (— Signal specific of saturated tetrasaccharides)	S-9
Figure S10: Chromatogram on AS11 (accelerated gradient) of the purified NRE building blocks <u>IIs$_{glu}$</u>^{sulf} (b) from sulfanilic tagged disaccharide fraction of porcine heparin digest (heparinase 1) (a)	S-10
Figure S11: Semi preparative chromatogram on AS11 (column 250x21mm) of the sulfanilic tagged porcine heparin digest (hep I) disaccharide fraction (— Signal specific of saturated tetrasaccharides)	S-11
NMR characterization	S-11
Table S3: Proton and carbon chemical shifts for IdoA2S-GlcNS6S (Is$_{id}^{sulf}$)	S-11

Figure S12: ^{1}H spectrum of Is_{id}^{sulf} (D2O, 25°C, 500 MHz)	S-12
Table S4: Proton and carbon chemical shifts for GlcA-GlcNS3S6S ($\underline{IIs_{glu}}^{sulf}$)	S-12
Figure S13: ^{1}H spectrum of $\underline{IIs_{glu}}^{sulf}$ (D2O, 25°C, 500 MHz)	S-13
Table S5: Proton and carbon chemical shifts for $GlcNS6S^{sulf}$	S-13
Figure S14: ^{1}H spectrum of GlcNS,$6S^{sulf}$ (D2O, 25°C, 500 MHz)	S-14
Figure S15: Chromatogram on Hypercarb of the $Mw515^{sulf}$ fraction the AS11 separation from figure S11 starting from digested PMH	S-15
Figure S16: ^{1}H spectrum of GlcA-GlcNS,6S ^{sulf} (IIs_{glu}^{sulf}) (D2O, 25°C, 500 MHz)	S-15
Table S6: Proton and carbon chemical shifts for GlcA-GlcNS,6S ^{sulf} (IIs_{glu}^{sulf})	S-16
Figure S17: ^{1}H spectrum of IdoA-GlcNS,6S ^{sulf} (IIs_{id}^{sulf}) (D2O, 25°C, 500 MHz)	S-16
Table S7: Proton and carbon chemical shifts for IdoA-GlcNS,6S ^{sulf} (IIs_{id}^{sulf})	S-17
**Identification of building blocks**	S-17
Figure S18: Ion-pair LC/MS of the sulfanilic tagged Semuloparin tetrasaccharide fraction and influence of the addition of β-glucuronidase. Chromatographic conditions : Identical to the description of the experimental part with a modified gradient (t0 %B0, t10min %B 25, t40min %B 80)(—UV 265 nm; — RIC m/z 1673.03 (Mw 1172^{sulf}); — RIC m/z 1593.13 (Mw 1092^{sulf}); — RIC m/z 1612.16 (Mw 996^{sulf}); — RIC m/z 1417.2 (Mw 916^{sulf})	S-17
Figure S19: Influence on the ion-pair chromatogram of the addition of β-D-glucuronidase to the heparinase digest of porcine heparin with sulfanilic tagging. a) UV 265nm b): NRE glucosamines + NRE disaccharides RIC m/z 415.1+495.06+575.05 +671.1+751.1	S-18
Figure S20 Influence on the ion pair chromatogram of the addition of Δ4-5-glycuronidase to the heparinase sulfanilic digest of heparin. (—232nm; —265nm)	S-19
**Optimization of chromatographic conditions on the AS11 method**	S-20
Table S8: Influence of the pH of mobile phase A on the retention time of building blocks	S-20
Figure S21: Influence of the pH of mobile phase A on the retention time of building blocks in the AS11 method	S-21
**Quantification of building blocks**	S-22
Figure S22: Heparin batch depolymerized by heparinase mixture and molecular weights applied for the quantification	S-22
Figure S23: Integration of the chromatograms (Figure 9A) due to digested porcine heparin	S-23
Table S9: Integration of peak areas determined from Figure 6 and integration results.	S-24
**Building blocks analysis with the classical method**	S-25
Table S10: Quantification of building blocks (% w/w) for PMH, OMH and BMH from Table 2 by the classical method [6]	S-27
**NMR comparison of heparins**	S-28
Figure S24: ^{1}H spectra of porcine, bovine and ovine mucosa heparins (D2O, 25°C, 800 MHz)	S-28

Isolation and identification of NRE tetrasaccharides in semuloparin fraction

Figure S1 shows the separation on AS11 of the tetrasaccharide fraction of semuloparin after reductive amination by sulfanilic tagging. The 2 NRE tetrasaccharides to be identified are easily detected by the selective UV signal (265 nm – 2.3 x 232 nm).

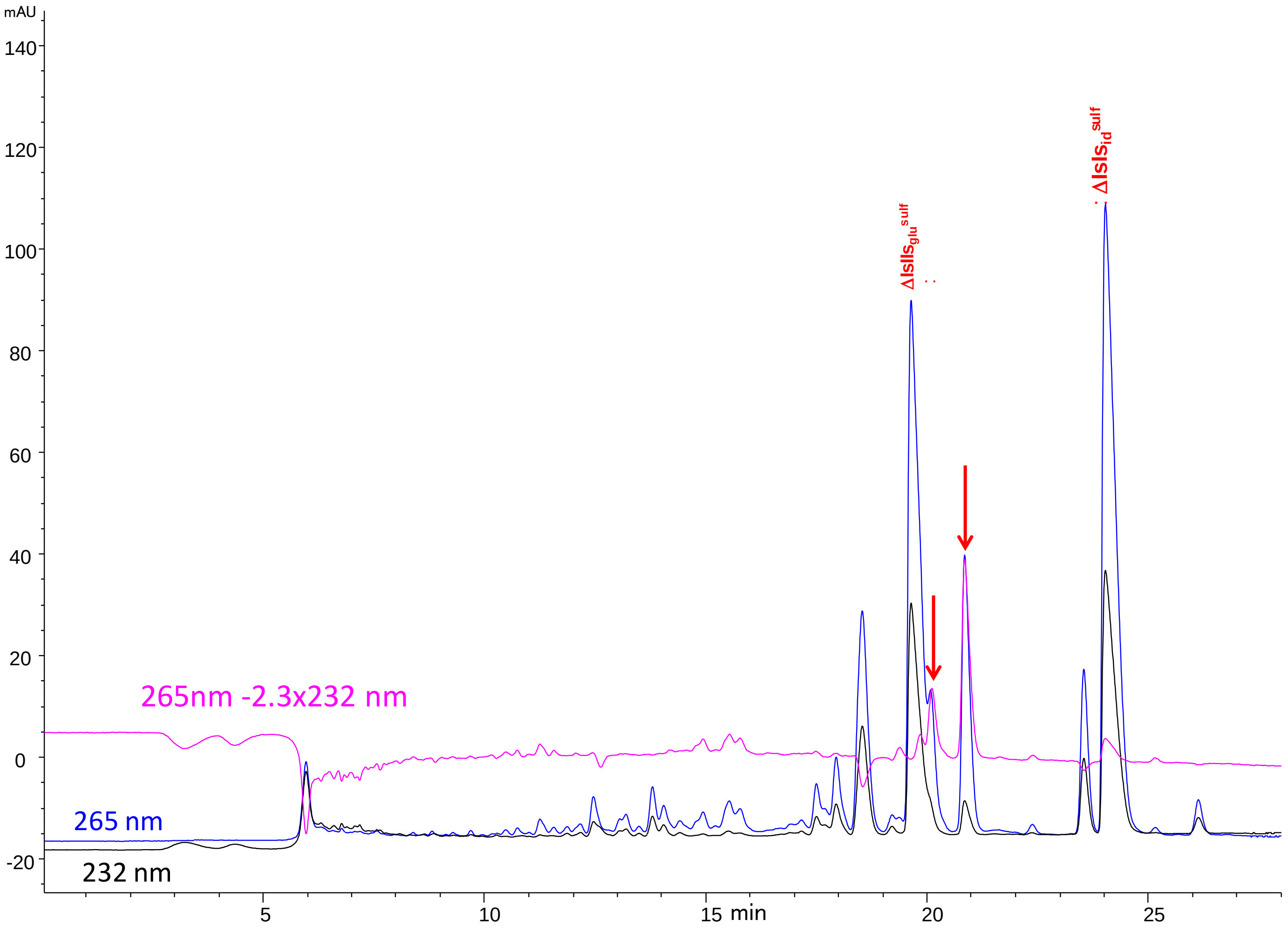

Figure S1: Chromatogram on AS11 (accelerated gradient) of the of sulfanilic tagged Semuloparin tetrasaccharide fraction (— Signal specific of saturated tetrasaccharides)

The semi preparative separation was done on AS11 column (250 x 11 mm) as described in the experimental part. 10 to 20 mg were injected. After the separation, the fractions were neutralized by addition of diluted ammonia and controlled on analytical AS11 (experimental conditions of figure S1). The selectivity between NRE tetrasaccharides and ◎Is-IIs$_{glu}$ was pH dependent. pH 2.5 was chosen for the semi preparative separation (figure S2).

The chromatograms of the 2 isolated tetrasaccharides after desalting on Sephadex G10 is shown in figure S3.

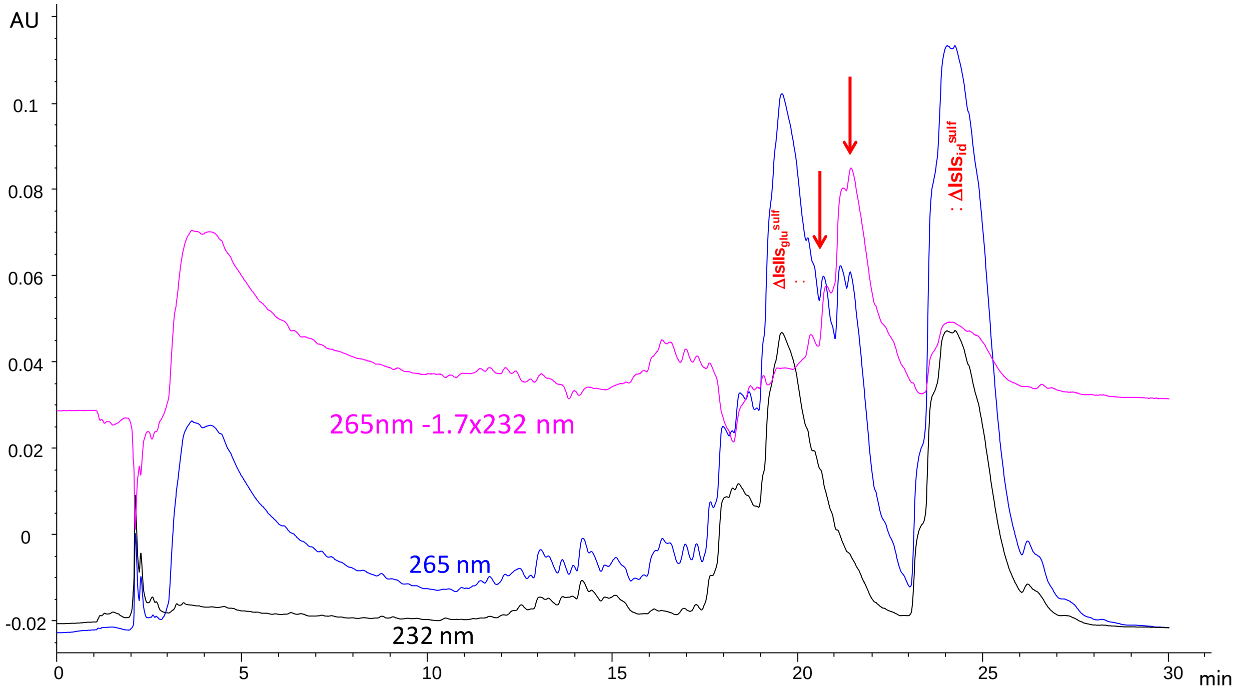

Figure S2: Semi preparative chromatogram on AS11 (column 250 x 21 mm) of the sulfanilic tagged Semuloparin tetrasaccharide fraction (— Signal specific of saturated tetrasaccharides)

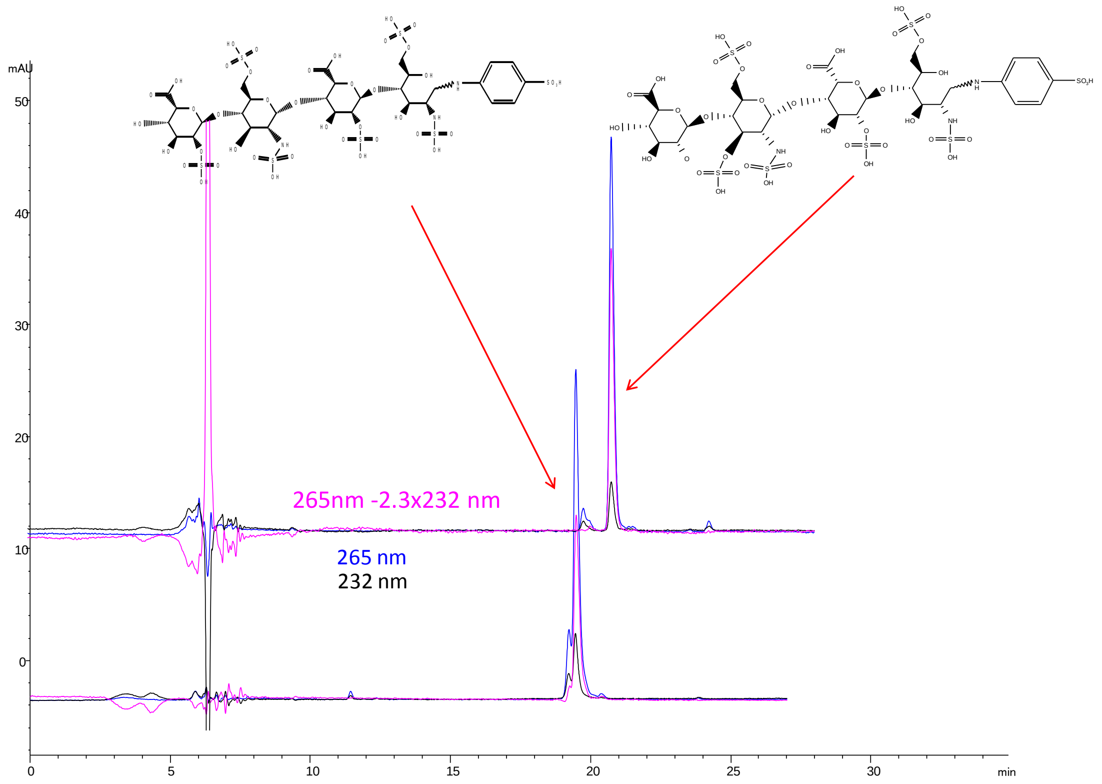

Figure S3: Chromatogram on AS11 of the 2 purified sulfanilic tagged NRE tetrasaccharides (— Signal specific of saturated tetrasaccharides)

NMR characterization

The structure of the main NRE tetrasaccharide could be identified directly by NMR in the tetrasaccharide semuloparin fraction.

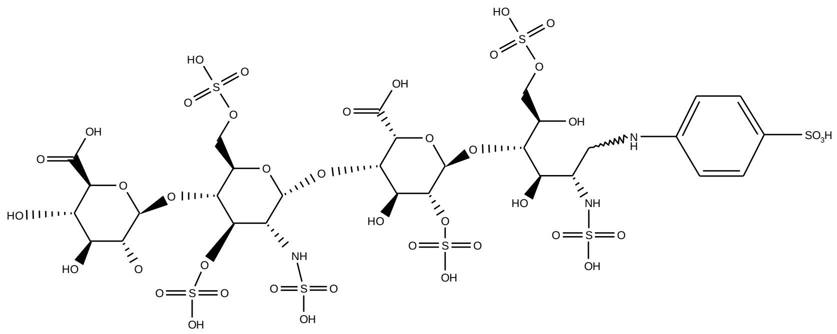

Figure S4: Structure of the tetrasaccharide <u>IIs_glu</u>-Is_id^sulf

$^1H/^{13}C$	**GlcA^{IV}**	**Glc2NS3S6S^{III}**	**IdoA2S^{II}**	**Glc2NS6S^{I}**
**1**	4.626/103.13	5.552/98.18	5.343/102.27	3.478-3.414/46.38
**2**	3.400/75.10	***3.476/58.74***	**4.434/79.26**	***3.605/55.60***
**3**	3.533/77.41	**4.396/78.51**	4.187/72.54	3.899/72.11
**4**	3.553/74.19	4.038/74.86	4.179/78.28	4.168/81.85
**5**	3.726/78.74	4.186/71.74	4.700/72.42	4.309/71.46
**6'6'**		**4.5061-4.281/68.15**		**4.223-4.151/70.37**
**Tosyl**				**NH**=8.460<br>7.843-7.605/114.73-129.19

Table S1: Proton and carbon chemical shifts for GlcA^{IV}-Glc2NS3S6S^{III}-IdoA2S^{II}-Glc2NS6S^{I} (<u>IIs_glu</u>-Is_id)^{sulf}

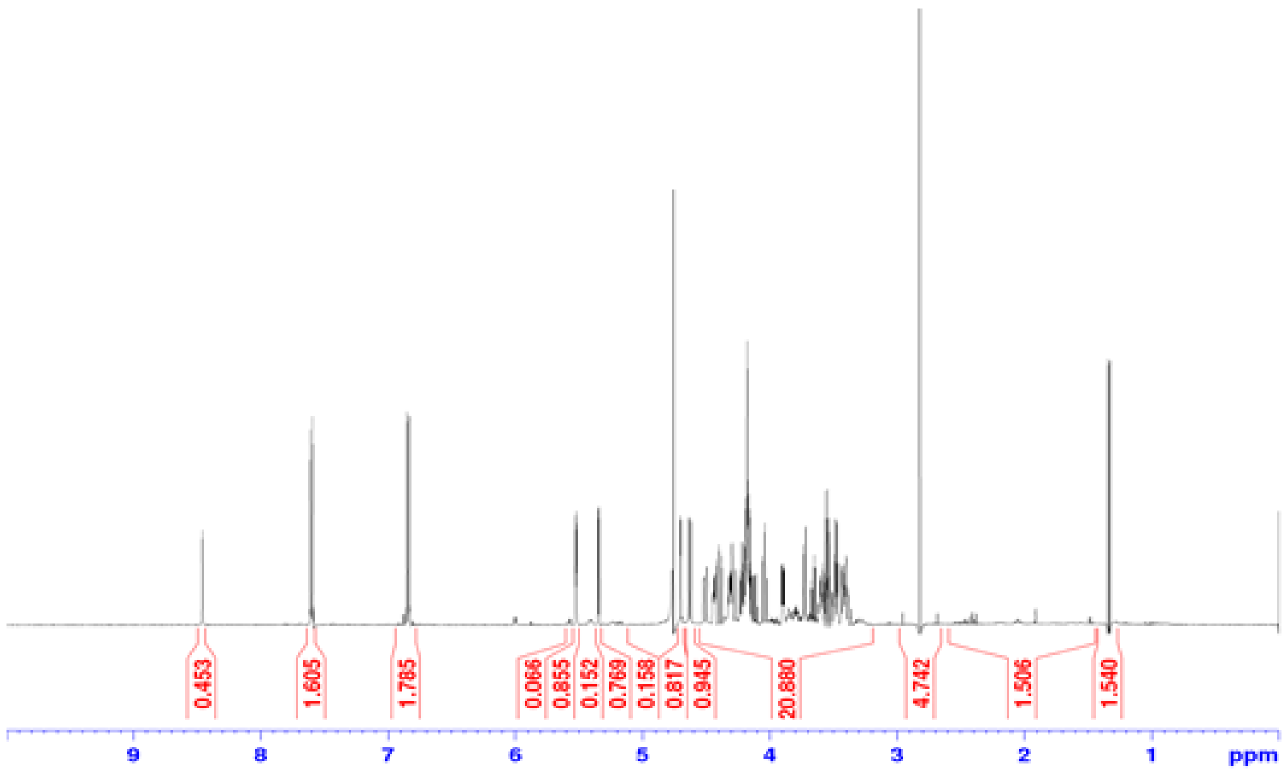

Figure S5: ^{1}H spectrum of $\underline{IIs_{glu}}$-Is_{id}^{sulf} (D2O, 25°C, 500 MHz)

The structure of the second minor NRE tetrasaccharide, determined by NMR is shown in figure S6.

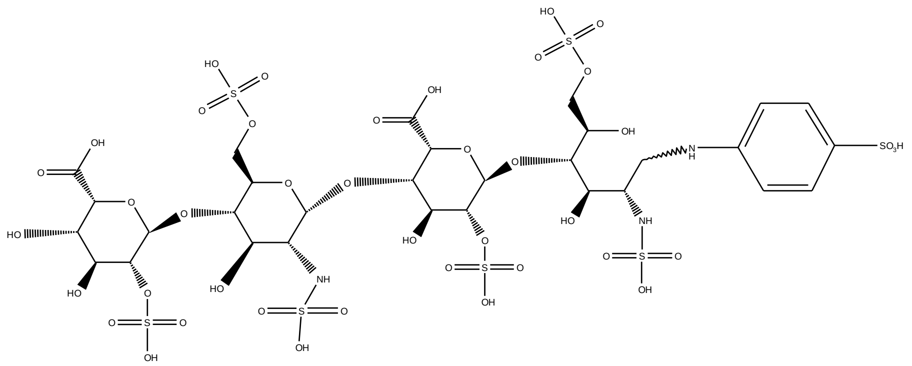

Figure S6: Structure of the minor NRE tetrasaccharide Is_{id}-Is_{id} after reductive amination by sulfanilic acid.

$\underline{IIs_{glu}}$-Is_{id} and Is_{id}-Is_{id} are the two NRE tetrasaccharides at 1172 Da detected by LC/MS in the fractions of semuloparin and enoxaparin from PMH.

$^{1}H/^{13}C$	**IdoA2S^{IV}**	**Glc2NS6S^{III}**	**IdoA2S^{II}**	**Glc2NS6S^{I}**
**1**	5.178/101.24	5.413/98.53	5.390/101.98	3.474-3.418/46.46
**2**	**4.313/76.06**	***3.275/60.10***	**4.431/78.17**	***3.587/55.44***
**3**	4.108/70.92	3.704/71.62	4.199/71.38	3.885/72.11
**4**	3.991/71.17	3.770/78.73	4.126/78.16	4.147/82.29
**5**	4.851/70.89	4.057/71.24	4.699/71.60	4.308/71.43
**6′6′**		**4.327-4.275/68.19**		**4.207-4.121/70.32**
**Tosyl**				**NH**=8.455<br>7.838-7.600/114.67-129.18

Table S2: Proton and carbon chemical shifts for IdoA2S^{IV}-Glc2NS6S^{III}-IdoA2S^{II}-Glc2NS6S^{I} (Is_{id}-Is_{id})^{sulf}

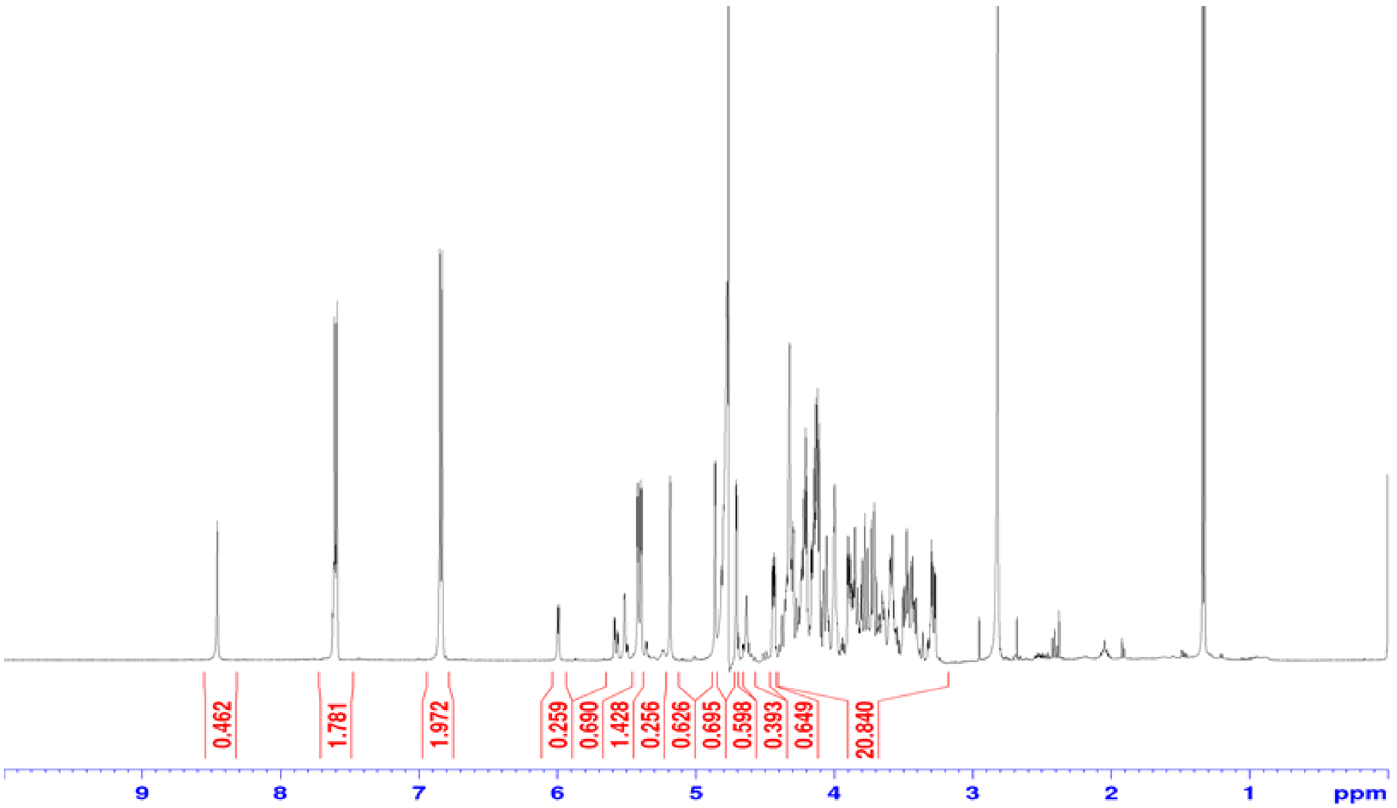

Figure S7: ^{1}H spectrum of $Is_{id}Is_{id}{}^{sulf}$ (D2O, 25°C, 500 MHz)

Isolation and identification of NRE disaccharides in disaccharide fractions from digested heparins

Disaccharide fractions from heparin digested by heparinase 1 were the easiest starting material to achieve the purification of the two NRE disaccharides Is_{id}^{sulf} and $\underline{IIs_{glu}}^{sulf}$.

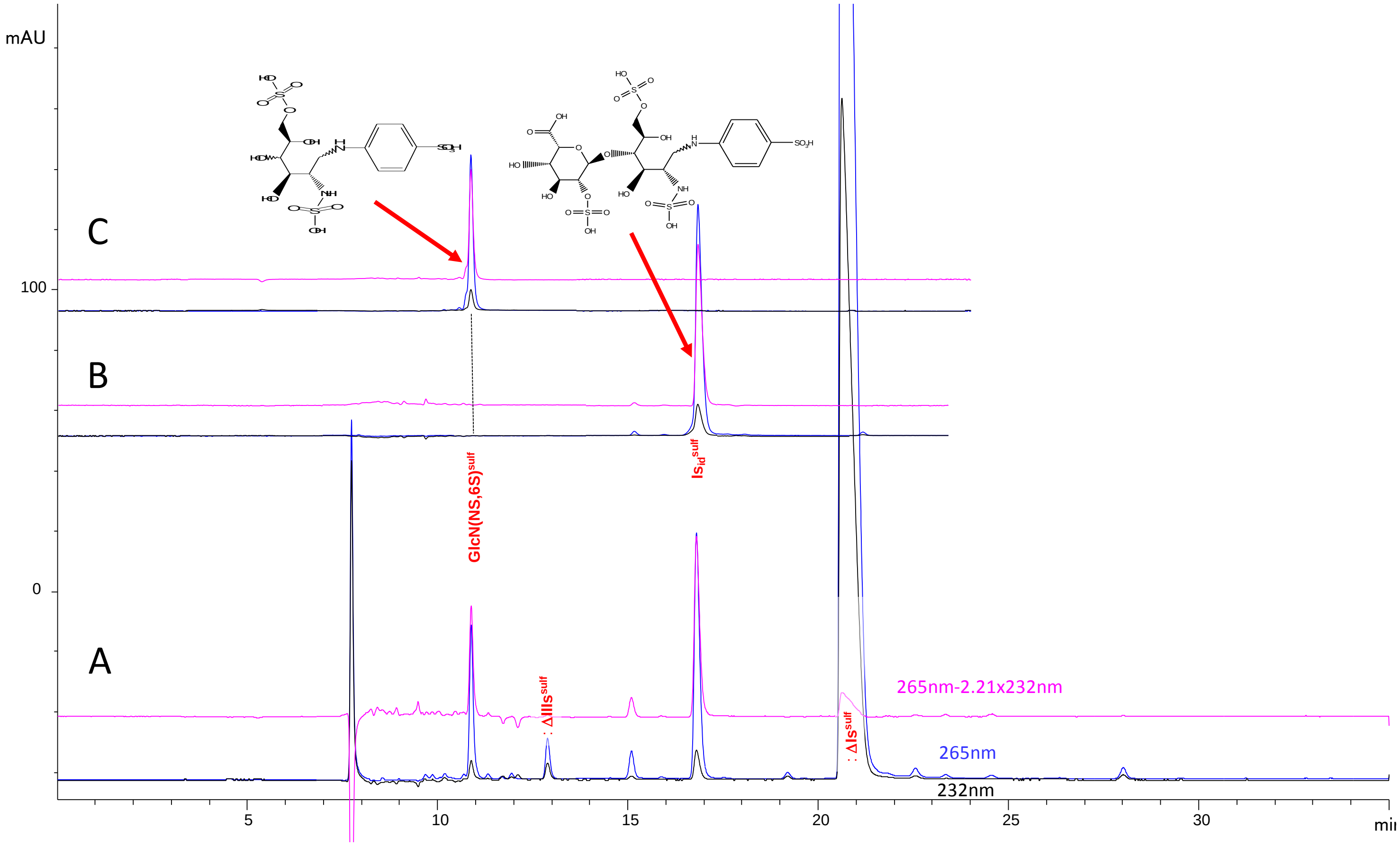

Figure S8: Chromatogram on AS11 (accelerated gradient) of the purified NRE building blocks Is_{id}^{sulf} (B) and $GlcN(NS,6S)^{sulf}$ (C) from sulfanilic tagged disaccharide fraction of ATIII low affinity fraction of BLH digest (heparinase 1) (A)

The ATIII low affinity fraction of BLH was chosen to purify Is_{id}^{sulf} (Figure S8), since none of the usual neighbouring building blocks $\underline{IIs_{glu}}^{sulf}$, $\Delta IIa\text{-}\underline{IVs_{glu}}^{sulf}$ or $GlcN(NS,3S,6S)^{sulf}$ are present in its disaccharide fraction. The purification was done on AS11 semi preparative column (Figure S9) in experimental conditions identical to those used for semuloparin tetrasaccharides except the pH of mobile phases which was set at 3

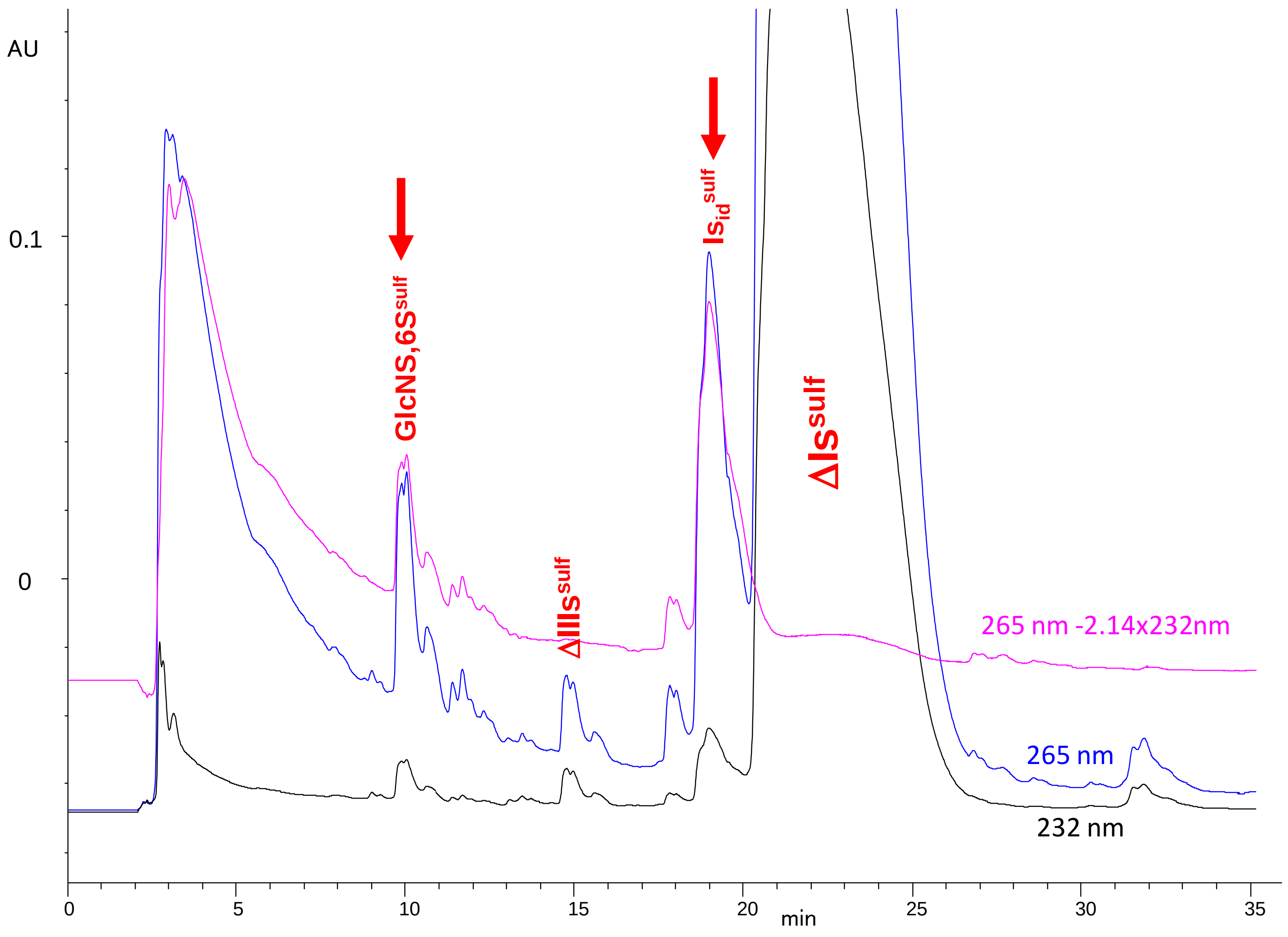

Figure S9: Semi preparative chromatogram on AS11 (column 250 x 21 mm) of the sulfanilic tagged BLH digest (hep I) disaccharide fraction (— Signal specific of saturated tetrasaccharides)

<u>IIs_{glu}^{sulf}</u> was isolated from the disaccharide fraction of PMH digested by heparinase 1 (Figure S10). The purification was realised on semi preparative AS11 column (Figure S11) as previously with mobiles phases pH adjusted at 2.5. Two other NRE disaccharides (Mw 515^{sulf}), IIs_{glu}^{sulf} and IIs_{id}^{sulf} were also further identified.

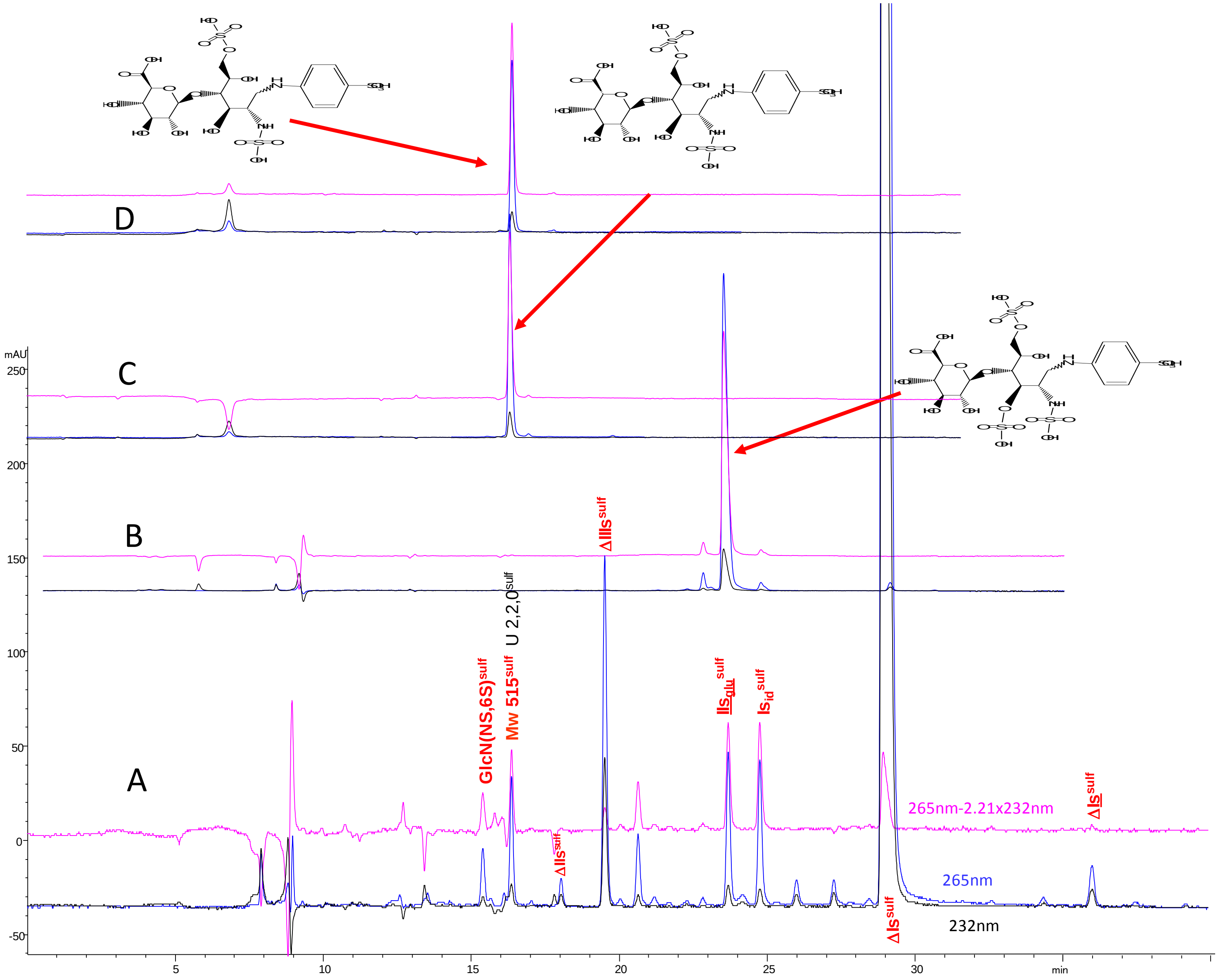

Figure S10: Chromatogram on AS11 (accelerated gradient) of the purified NRE building blocks IIs_{glu}^{sulf} (D), IIs_{id}^{sulf} (C), <u>IIs</u>$_{glu}^{sulf}$ (B) from sulfanilic tagged disaccharide fraction of PMH digest (heparinase 1) (A)

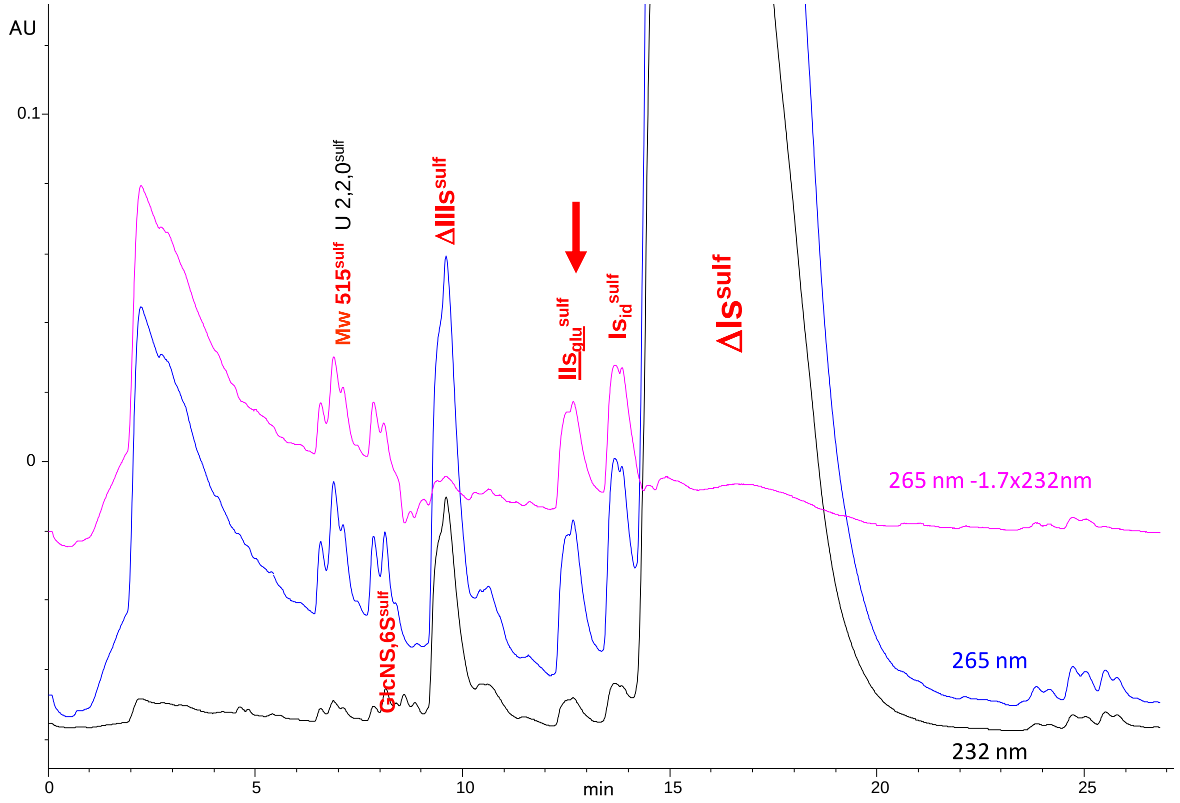

Figure S11: Semi preparative chromatogram on AS11 (column 250 x 21mm) of the sulfanilic tagged PMH digest (hep I) disaccharide fraction (— Signal specific of saturated tetrasaccharides)

NMR Characterization

	**IdoA[2] αL $^3J_{H1H2}$ = 1.6 Hz**	**GlcNS[1]**
**1**	5.327/102.41	3.499-3.420/47.14
**2**	**4.361/77.53**	**3.630/56.43**
**3**	4.043/72.27	3.927/73.05.
**4**	3.983/72.33	4.144/82.80.
**5**	4.633/72.21	4.332/72.20
**CH_2 66′**		**4.239-4.128/71.28**
**Tosyl**		7.600-6.840/129.88-115.41

Table S3: Proton and carbon chemical shifts for IdoA2S-GlcNS6S (Is_{id}^{sulf})

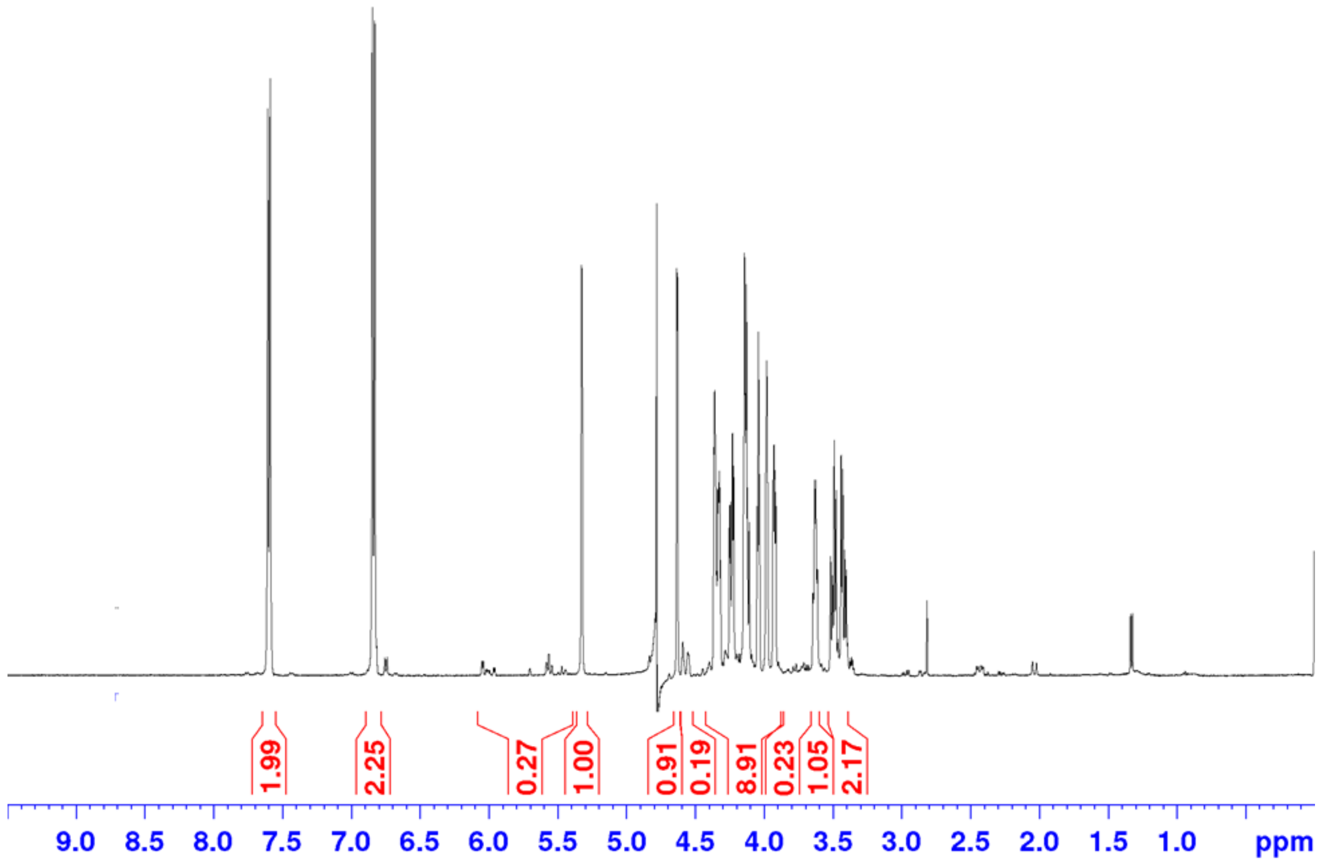

Figure S12: ^{1}H spectrum of Is_{id}^{sulf} (D2O, 25°C, 500 MHz)

	GlcA[2]	GlcNS[1]
**1**	4.639/104.68	3.736-3.592/45.47
**2**	3.198/76.28	**4.074/54.76**
**3**	3.497/78.15	**4.956/80.16**
**4**	3.481/74.86	4.353/77.11.
**5**	3.727/78.67	4.229/71.65
**CH2 66'**		**4.338-4.274/71.72**
**Tosyl**		7.613-6.880/129.74-115.98

Table S4: Proton and carbon chemical shifts for GlcA-GlcNS3S6S (IIs_{glu}^{sulf})

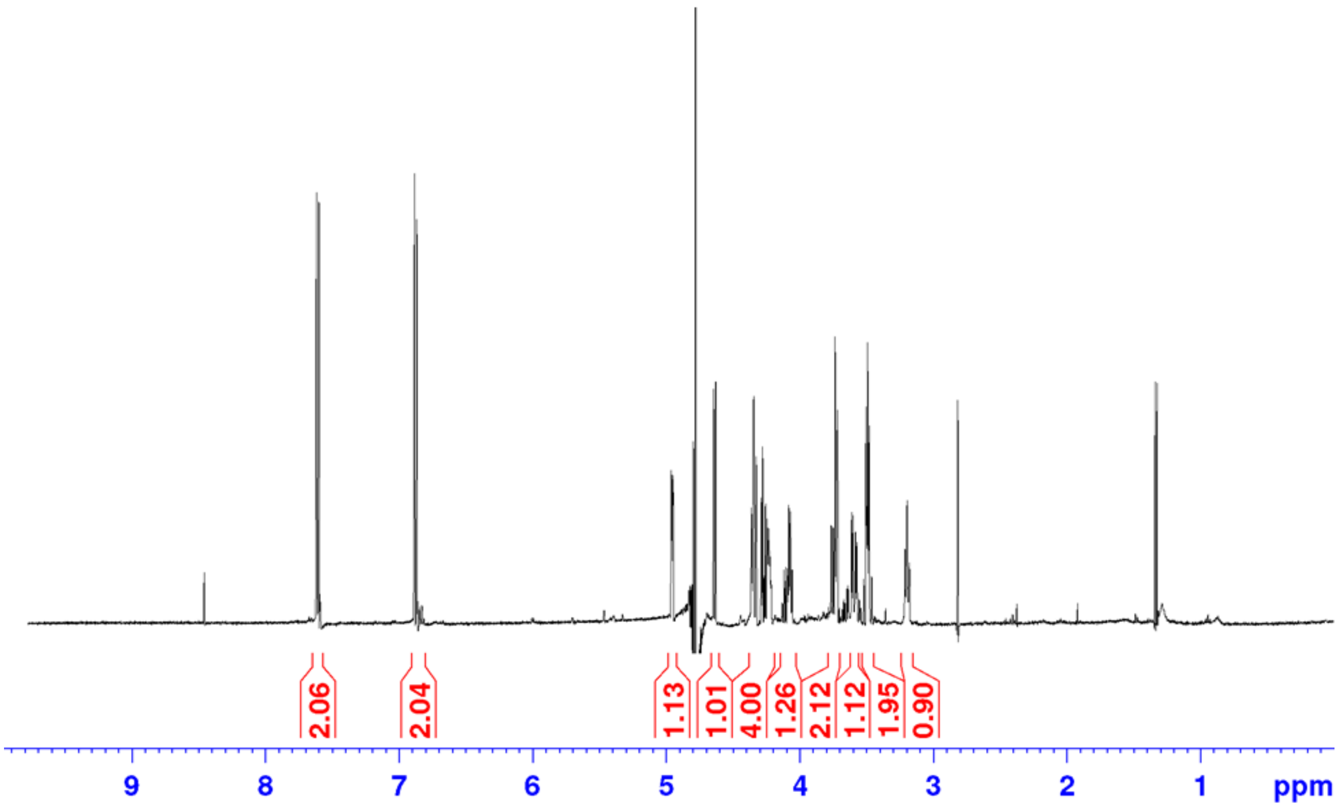

Figure S13: ^{1}H spectrum of $\underline{IIs_{glu}}^{sulf}$ (D2O, 25°C, 500 MHz)

	**GlcNS[1]**
**1**	3.517-3.333/47.17
**2**	**3.648/58.37**
**3**	4.000/71.61.
**4**	3.808/73.96.
**5**	3.973/71.99
**CH2 66′**	**4.298-4.141/72.62**
**Tosyl**	7.614-6.850/129.95-115.57

Table S5: Proton and carbon chemical shifts for GlcNS6S^{sulf}

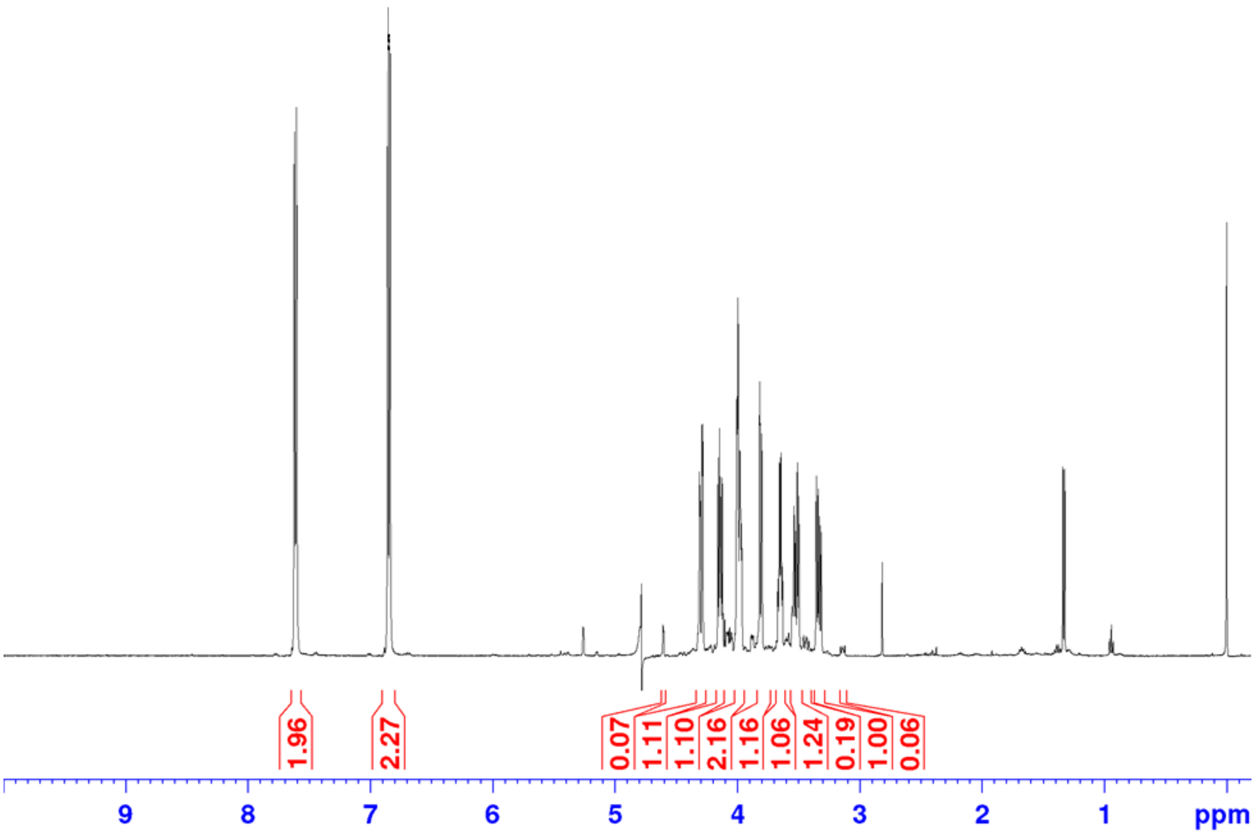

Figure S14: ^{1}H spectrum of GlcNS,6S^{sulf} (D2O, 25°C, 500 MHz)

The purification of the NRE disaccharides at Mw 515^{sulf} obtained from digested PMH (Figure S11) was realized by injecting the fraction collected from the AS11 semi preparative step on 2 Hypercarb columns (Thermoscientific) (150 x 10 mm) (5 µm particle diameter) connected in series (Figure S15). Solvent A was 50 mM $NaClO_4$ set at pH 2.88 by addition of perchloric acid. Solvent B was CH_3CN-H_2O 80-20v/v with 100 mM $NaClO_4$; pH3. A linear gradient (t0 min B% 0; t 25 min B% 20; t 40 min B% 40) was applied for the elution at a flow rate of 4 mL/min. Separation was realized at room temperature and UV detection was used with a diode array detector. The 2 disaccharides IIs_{glu}^{sulf} and IIs_{id}^{sul} were isolated. Their NMR ^{1}H spectra with full NMR determination are on figures S16 and S17 and on table S6 and S7.

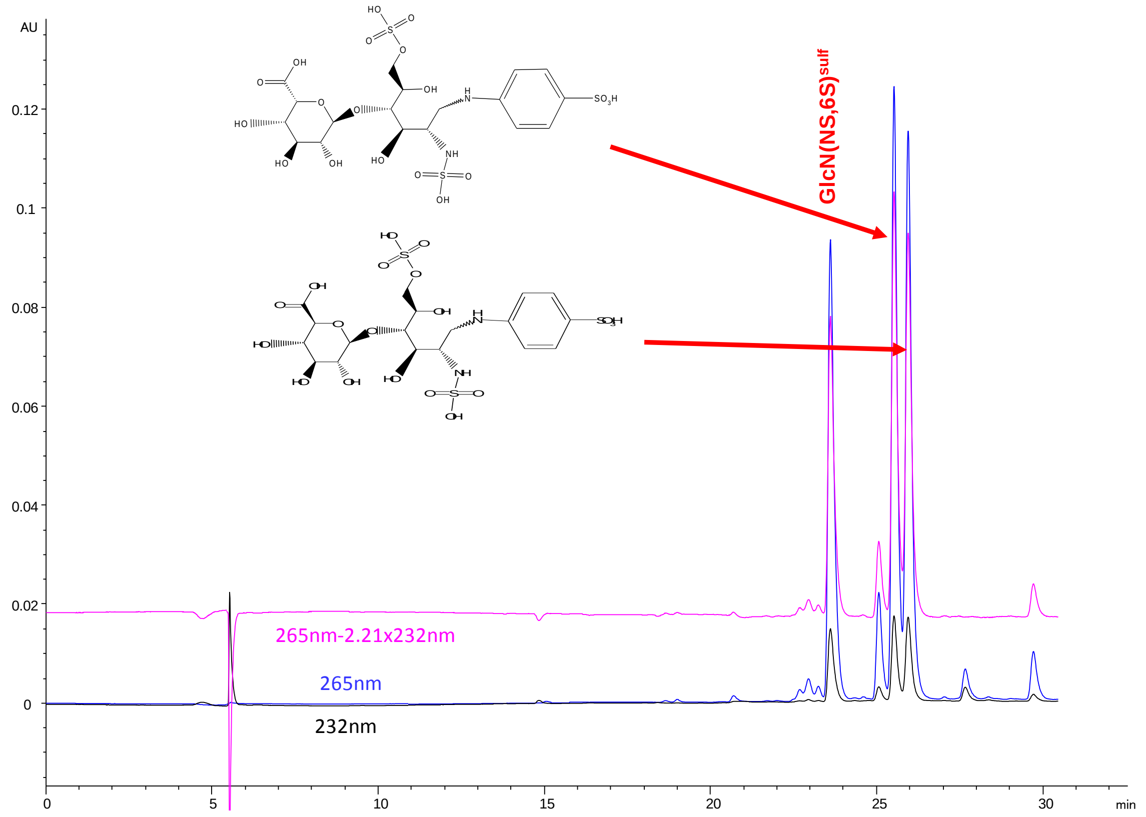

Figure S15: Chromatogram on Hypercarb of the Mw515^sulf fraction the AS11 separation from figure S11 starting from digested PMH

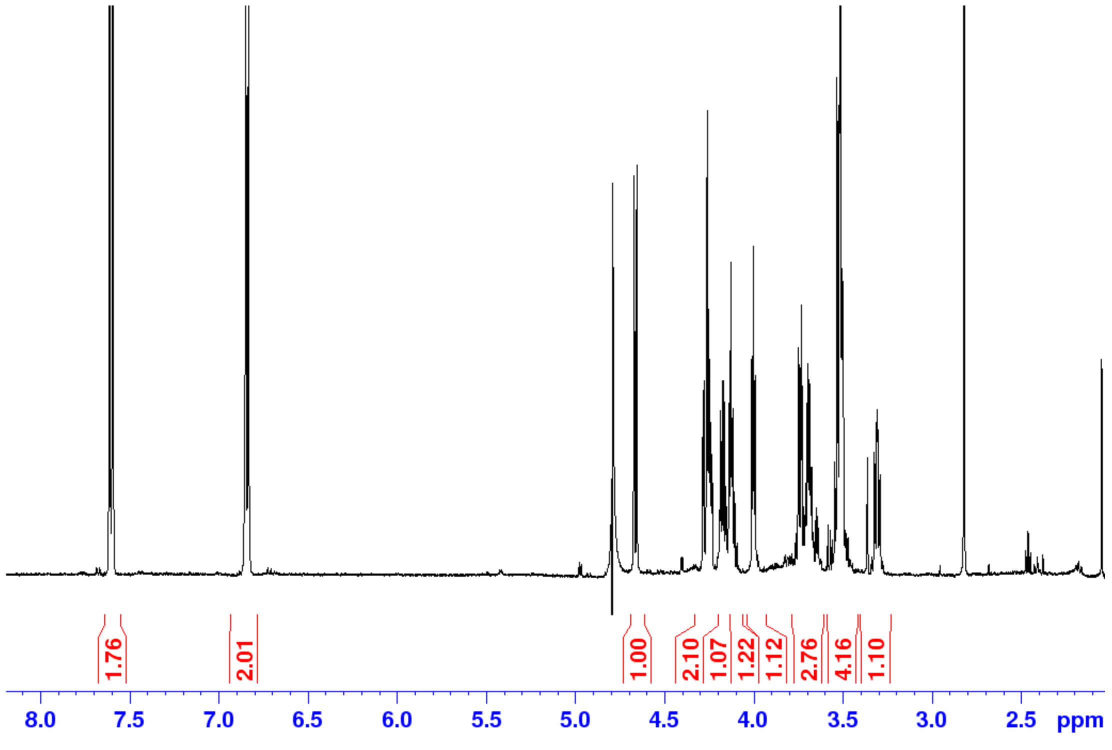

Figure S16: ^{1}H spectrum of GlcA-GlcNS,6S ^{sulf} (IIs_{glu}^{sulf}) (D2O, 25°C, 500 MHz)

	GlcA[2]	GlcNS[1]
**1**	4.658/102.59	3.495/43.76
**2**	3.301/73.31	**3.689/53.36**
**3**	3.508/75.41	3.993/69.77
**4**	3.508/71.79	4.123/68.49
**5**	3.735/76.02	4.242/69.39
**CH2 66′**		**4.166 - 4.263/68.48**
**Tosyl**		7.599 - 6.837/126.85 – 112.68

Table S6: Proton and carbon chemical shifts for GlcA-GlcNS,6S ^{sulf} (IIs$_{glu}^{sulf}$)

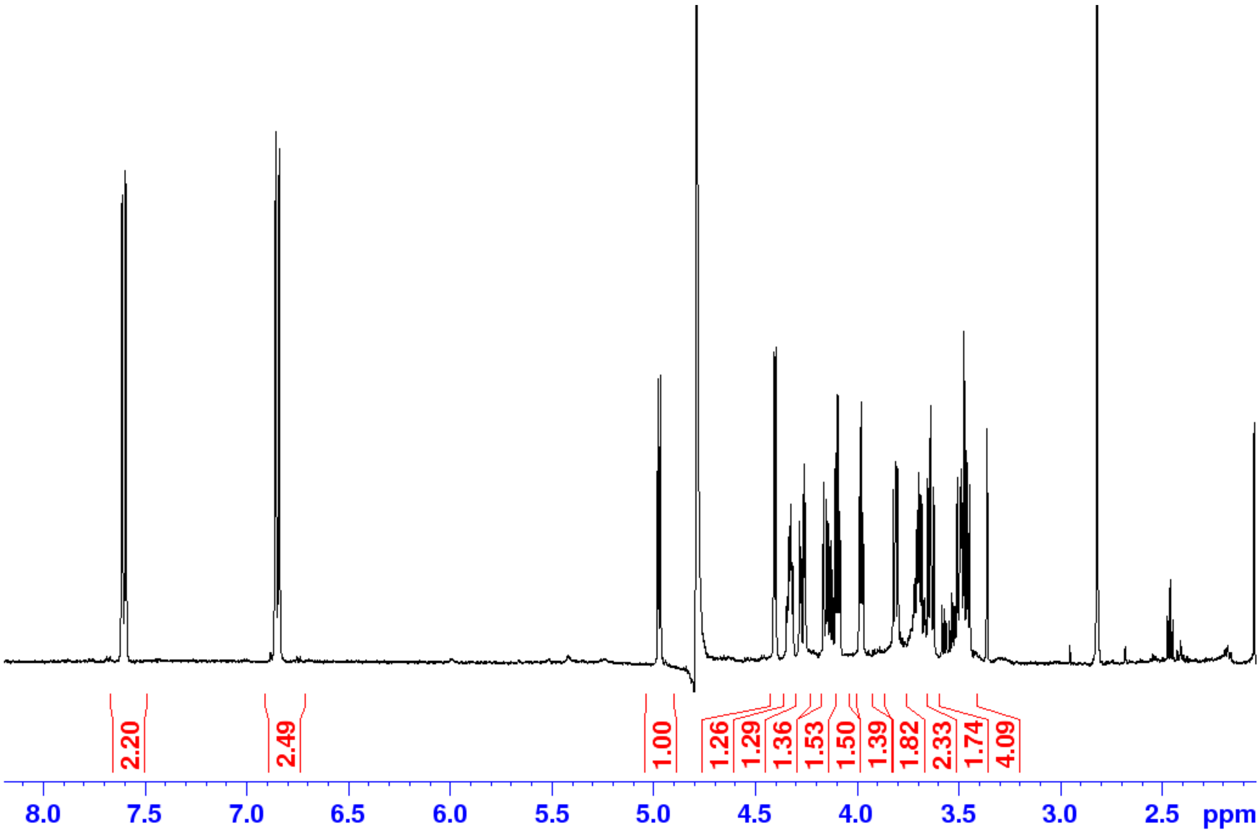

Figure S17: ^{1}H spectrum of IdoA-GlcNS,6S ^{sulf} (IIs$_{id}^{sulf}$) (D2O, 25°C, 500 MHz)

	IdoA²	GlcNS¹
**1**	4,966/ 101,4	3,483/43,93
**2**	3,466/71,48	**3,681/53,29**
**3**	3,632/72,68	3,974/70,26
**4**	3,806/71,35	4,091/78,92
**5**	4,395/71,71	4,324/69,40
**CH2 66′**		**4.150 - 4.265/68,63**
**Tosyl**		7,599 - 6,844/127,09 – 112,56

Table S7: Proton and carbon chemical shifts for IdoA-GlcNS,6S ^{sulf} (IIs_{id}^{sulf})

Identification of building blocks

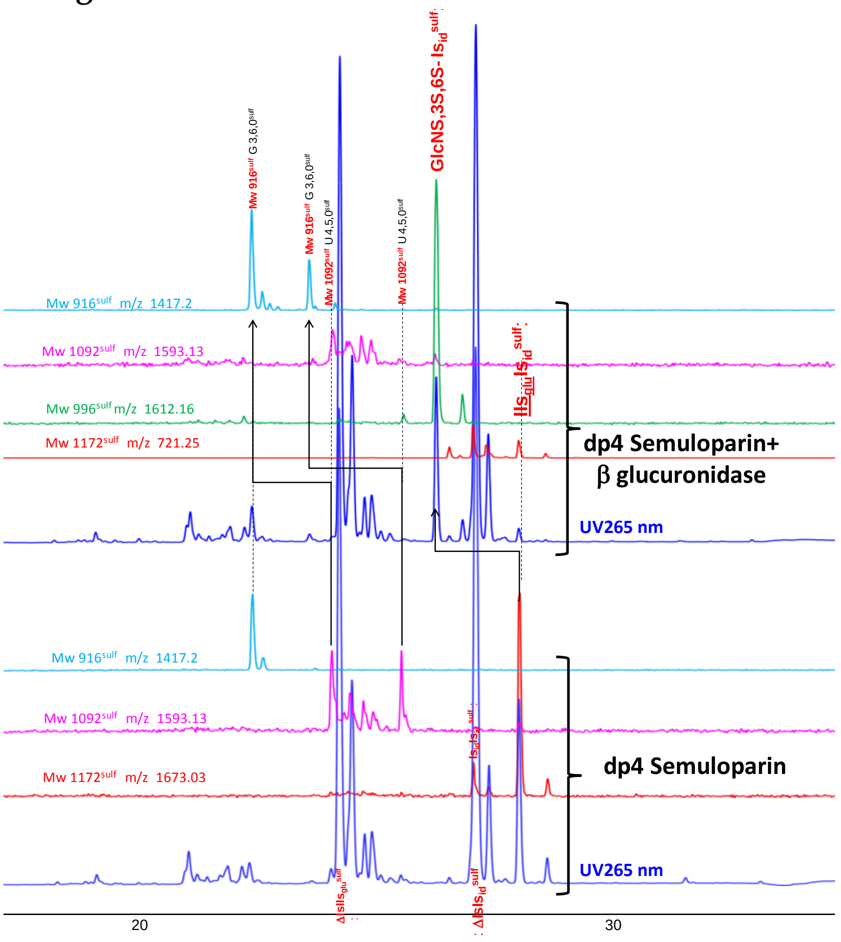

Figure S18: Ion-pair LC/MS of the sulfanilic tagged Semuloparin tetrasaccharide fraction and influence of the addition of β-glucuronidase. Chromatographic conditions: Identical to the description of the experimental part with a modified gradient (t0 %B0, t10min %B 25, t40min %B 80)(— UV 265 nm; — RIC m/z 1673.03 (Mw 1172^{sulf}); — RIC m/z 1593.13 (Mw 1092^{sulf}); — RIC m/z 1612.16 (Mw 996^{sulf}); — RIC m/z 1417.2 (Mw 916^{sulf})

Figure S18 shows the ion-pair chromatogram of the semuloparin tetrasaccharide fraction and the action of β-glucuronidase. Since the structure of the major NRE tetrasaccharide is $\underline{IIs_{glu}}Is_{id}^{sulf}$, β-glucuronidase cleaves the glucuronic acid and gives GlcNS,3S,6S-Is_{id}^{sulf} at Mw 996^{sulf}, in line with figure S18. The aim of this preliminary experiment was mainly to check that β-glucuronidase was active on sulfanilic tagged oligosaccharides to prepare the next step corresponding to the addition of β-glucuronidase to the porcine digest of figure 7 and figure S19. The action of β-glucuronidase in figure S18 also shows that 2 minor NRE tetrasaccharides at Mw 1092^{sulf} are cleaved resulting in 2 different trisaccharides at 916^{sulf}. One of this trisaccharides, corresponding probably to GlcNS,6S-Is_{id}^{sulf} eluted at 21.1 min is initially present in the fraction. The corresponding tetrasaccharide is likely $IIs_{glu}Is_{id}^{sulf}$. The second tetrasaccharide is less obvious to identify. The 6-O desulfation $\underline{IVs_{glu}}Is_{id}^{sulf}$ is possible but it seems difficult to exclude the possibility of a desulfated reducing disaccharide like $\underline{IIs_{glu}}IIIs_{id}^{sulf}$.

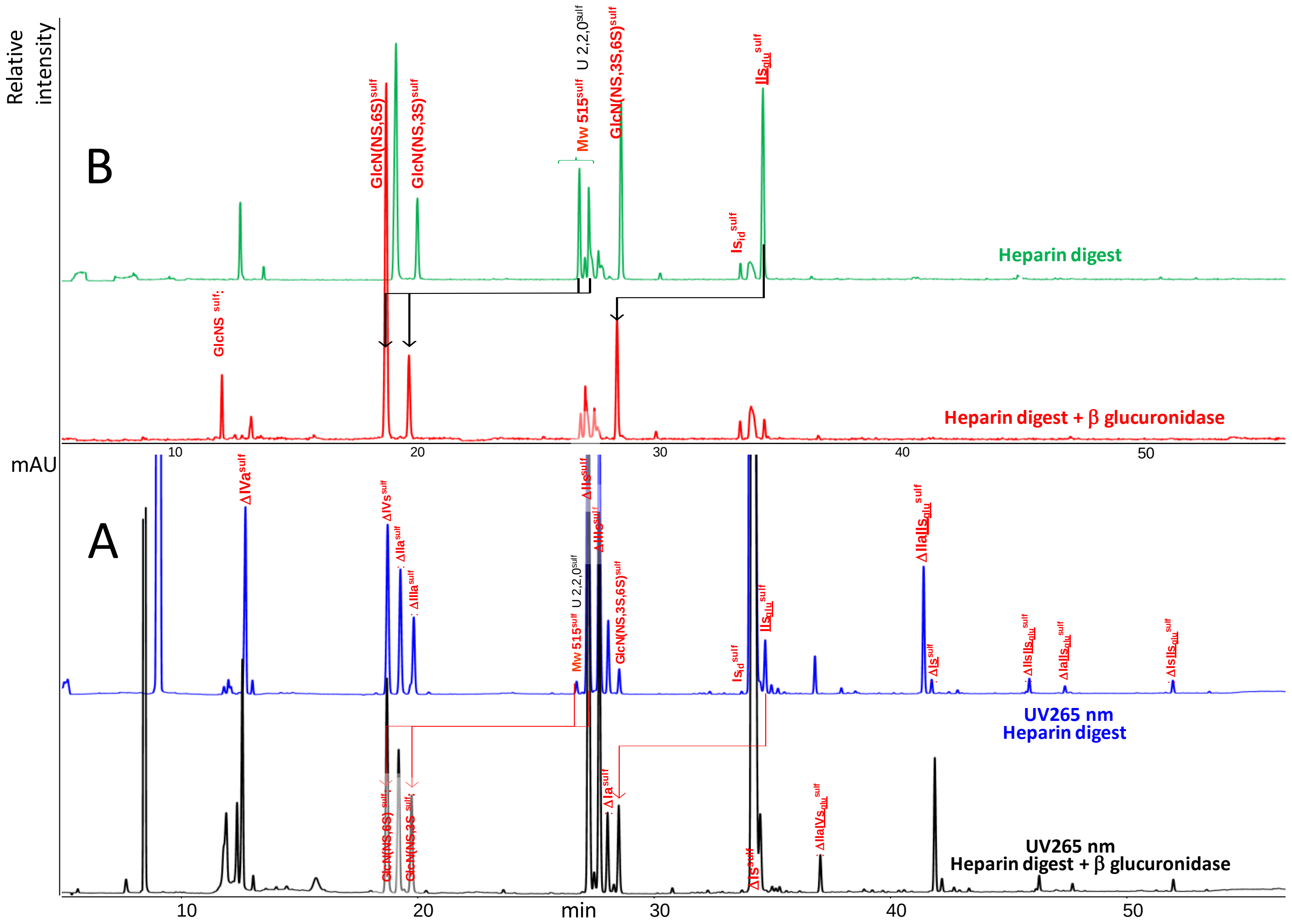

Figure S19: Influence on the ion-pair chromatogram of the addition of β-D-glucuronidase to the heparinase digest of PMH with sulfanilic tagging. A) UV 265nm B): NRE glucosamines + NRE disaccharides RIC m/z 415.1+495.06+575.05+671.1+751.1

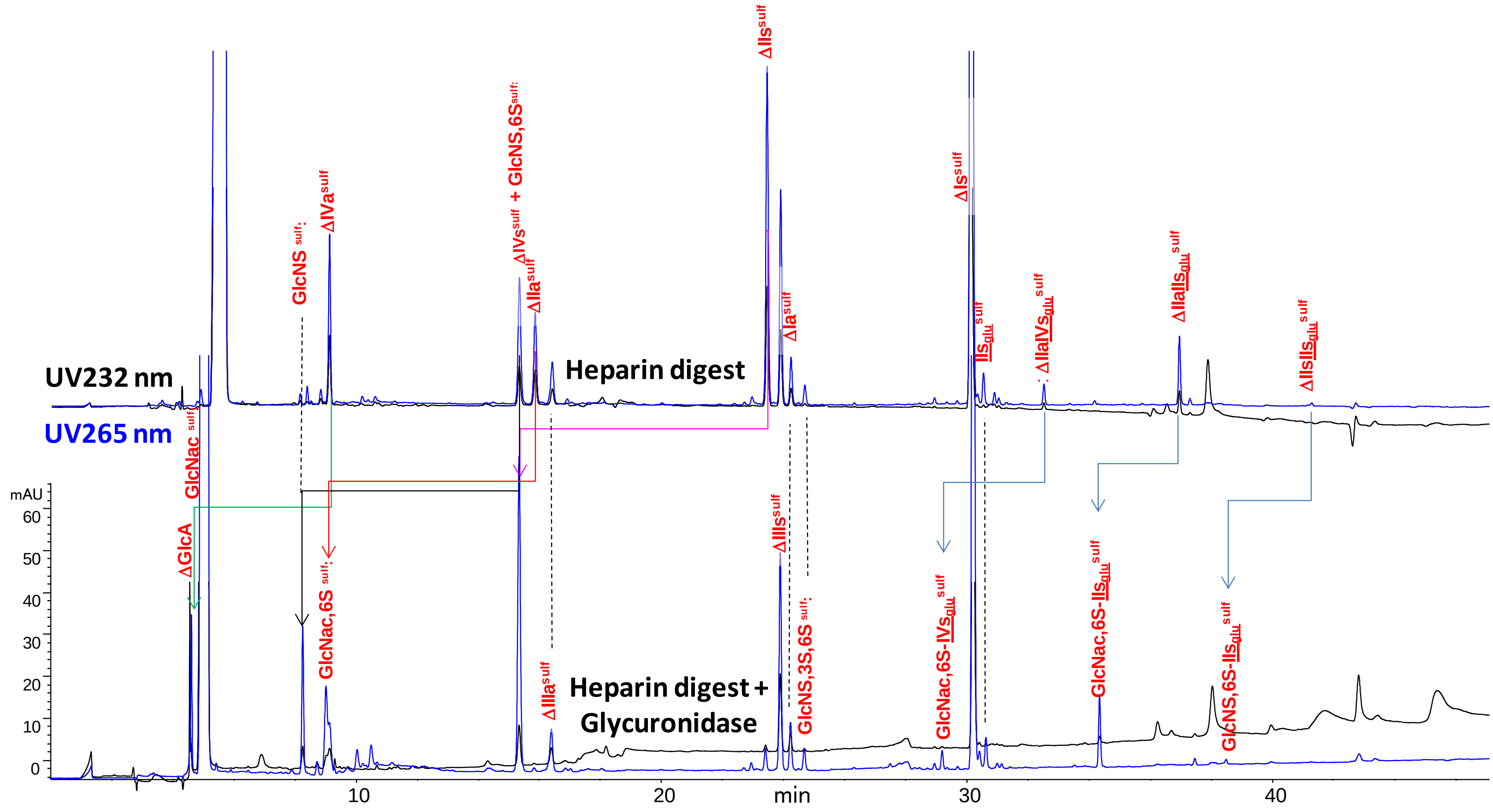

Figure S20 Influence on the ion pair chromatogram of the addition of Δ4-5-glycuronidase to the heparinase sulfanilic digest of heparin. (— 232 nm; — 265 nm)

The figure S20 is the equivalent of figure 8 with the AS11 method.

Optimization of chromatographic conditions on the AS11 method

The influence of the pH of mobile phase A (2.5 mM NaH_2PO_4) on a single AS11 column with the following gradient (t0 min %B 0; t 80min %B 60).

pH	2.5	2.7	2.95	3.2	Trend curves
ΔIVa	5.46	7.18	8.57	9.34	$y = -5.73x^2 + 38.2x - 54.18$
NRE(Glc(NS))	10.54	10.72	10.87	11.14	y = 0.83x + 8.46
ΔIVs$_{gal}$	12.37	13.19	14.01	15.27	y = 4.06x + 2.20
ΔIVs	12.59	13.44	14.30	15.58	y = 4.19x + 2.10
ΔIIa	13.33	14.12	14.93	16.12	y = 3.91x + 3.53
ΔIIIa	14.55	15.36	16.16	17.28	y = 3.83x + 4.98
NRE(GlcNS,3S)	19.18	19.48	19.62	19.92	y = x + 16.72
NRE(Glc(NS,6S))	19.18	19.48	19.78	20.11	y = 1.31x + 15.91
NRE(U(2,2,0))a	18.07	18.96	19.98	21.17	y = 4.39x + 7.08
NRE(U(2,2,0))	18.38	19.13	19.98	21.17	y = 3.93x + 8.51
ΔIIs$_{gal}$	19.68	20.67	21.44	22.65	y = 4.11x + 9.44
ΔIIs	19.68	20.67	21.72	23.02	y = 4.71 + 7.91
ΔIIIs	21.23	22.18	23.20	24.38	y = 4.45x + 10.11
ΔIa	23.12	23.97	25.01	26.07	y = 4.21x + 12.60
NRE(<u>IIs$_{glu}$</u>))	25.44	26.35	27.59	28.94	y = 5.00x + 12.88
ΔIIa-<u>IVs</u>	25.57	26.89	28.35	29.88	y = 6.12x + 10.32
NRE(Is$_{id}$))	26.97	27.76	28.75	29.89	y = 4.15x + 16.56
Δ<u>IIs</u>	27.57	28.31	29.24	29.89	y = 3.35x + 19.25
NRE(GlcNS,3S,6S)	29.03	29.4	29.84	30.16	y = 1.63x + 25.0
Δ<u>IIIs</u>	28.39	29	29.84	30.90	y = 3.57x + 19.40
NRE(G(3,4,0))	30.40	30.87	31.75	32.79	y = 3.44x + 21.68
ΔIs	30.31	31.38	32.56	33.56	y = 4.64x + 18.79
ΔIIa-<u>IIs</u>	32.50	33.87	35.28	36.80	y = 6.08x + 17.36
NRE(G(3,5,0))	37.87	38.8	39.81	40.84	y = 4.22x + 27.37
Δ<u>Is</u>	38.35	39.04	39.82	40.80	y = 3.46x + 29.68
ΔIIs-<u>IIs</u>	39.26	40.57	41.88	43.29	y = 5.69x + 25.10
ΔIa-<u>IIs</u>	40.62	41.88	43.10	44.39	y = 5.32x + 27.41
ΔIs-<u>IIs</u>	47.13	48.28	49.20	50.42	y = 4.58x + 35.77

Table S8: Influence of the pH of mobile phase A on the retention time (min) of building blocks

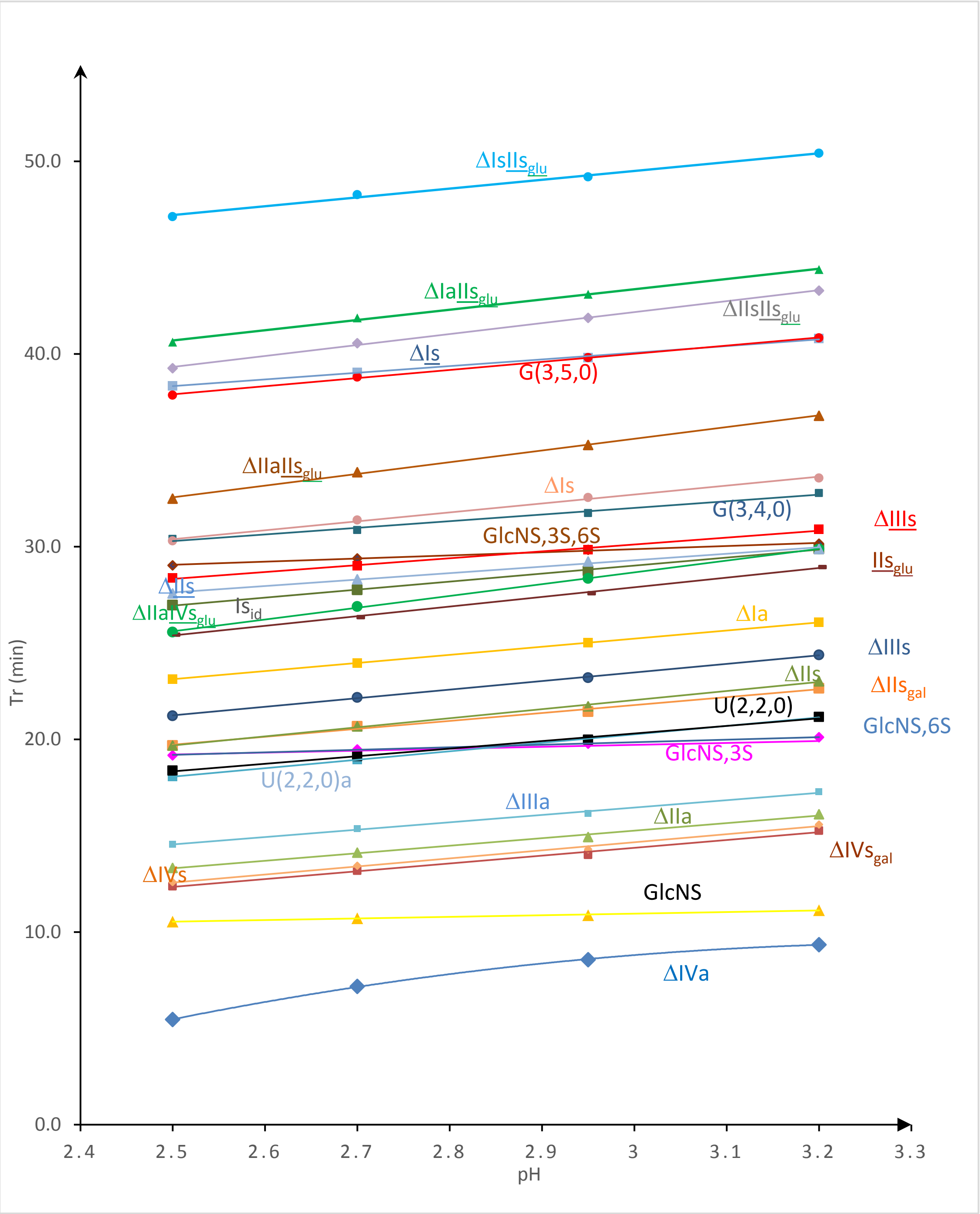

Figure S21: Influence of the pH of mobile phase A on the retention time of building blocks in the AS11 method

Quantification of building blocks

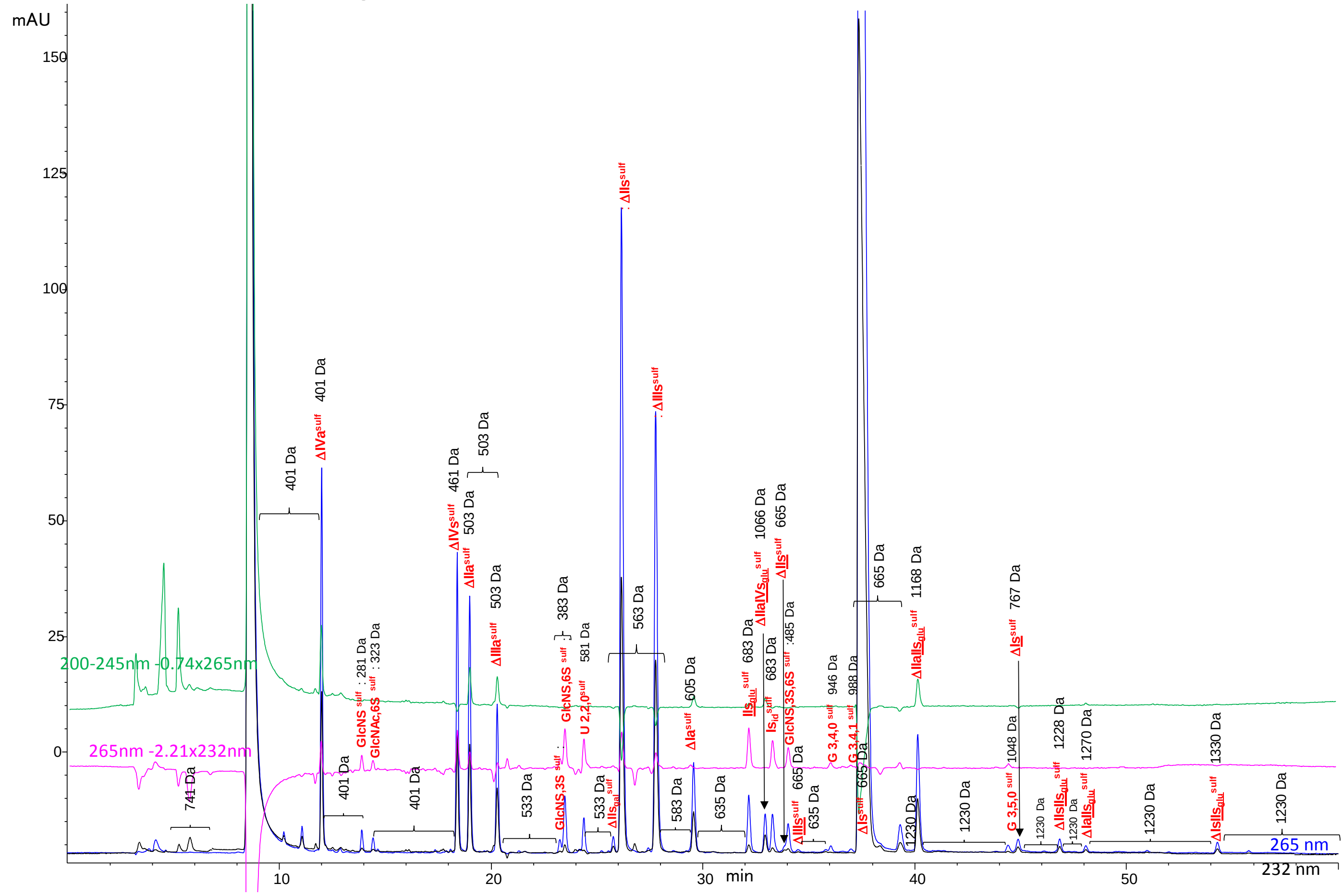

Figure S22: Heparin batch depolymerized by heparinase mixture and molecular weights applied for the quantification

Example of building block quantification for a heparin sample

The chromatograms of figure 9A corresponding to digested PMH is taken as example for the quantification. The chromatograms at 265 nm and 232 nm were integrated. The signal at 232 nm was integrated for tr<10min corresponding to glycoserines. Since they have no sulfanilic label, these areas are multiplied by 2.5, the response coefficient 265 nm/232 nm. Table S9 summarizes the peak areas, the identification of the peaks, and their molecular weights. The percentage (w/w) is obtained by the formula given in the main text.

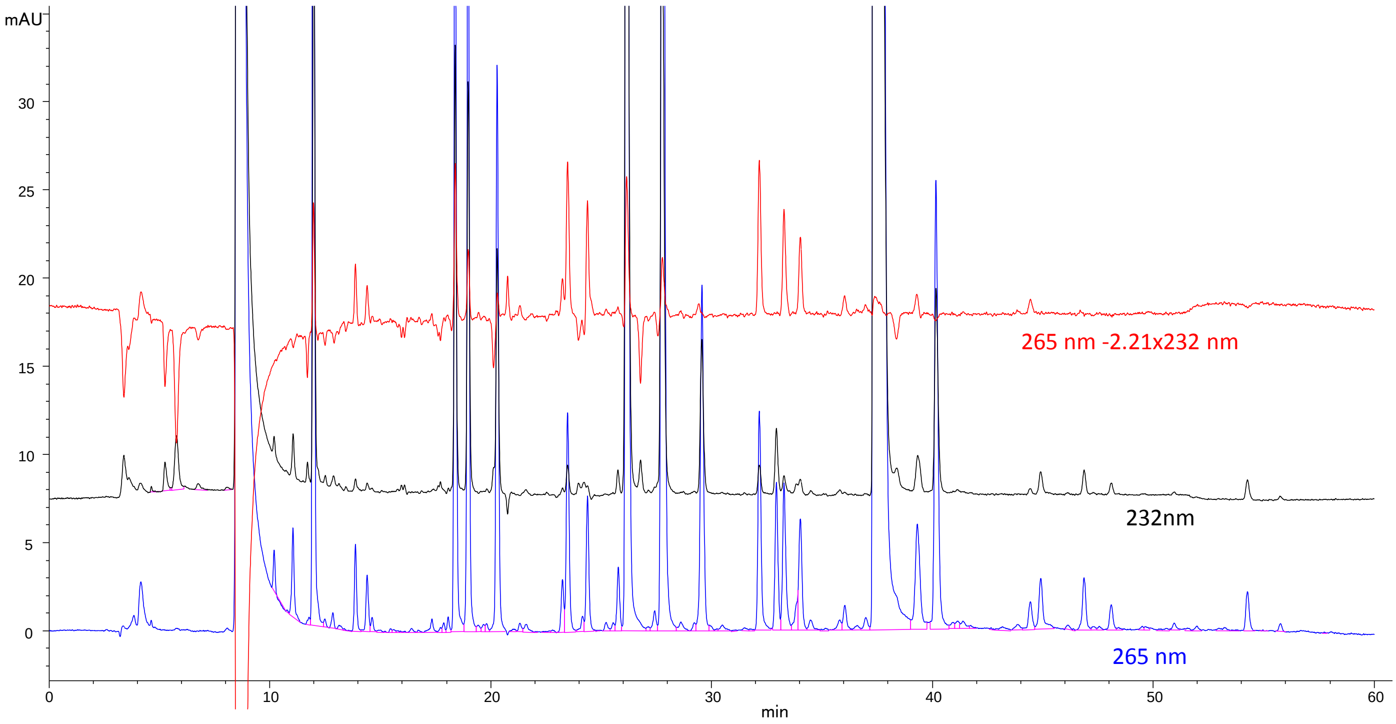

Figure S23: Integration of the chromatograms (Figure 9A) due to digested PMH

Time (Min)	Area (265 nm)	Name	Molecular weight Mw (Da)	Area x Mw	% (w/w)
4.6	4.04*		741	2993	
4.8	0.69*		741	511	
4.9	0.07*		741	27	
5.3	38.21*		741	28314	0.3
5.5	1.81*		741	1343	
5.8	97.63*		741	72347	0.8
6.8	11.50*		741	8518	
10.2	12.29		401	9106	
10.8	0.81		401	325	
11.1	38.75		401	15538	0.2
11.8	3.04		401	1219	
12.0	519.89	ΔIVa	401	208478	2.3
12.5	3.39		401	1360	
12.9	6.76		401	2709	
13.1	2.77		401	1110	
13.4	0.57		401	229	
13.6	0.23		401	90	
13.9	35.01	GlcNS	281	9839	0.1
14.4	26.39	GlcNAc6S	323	8524	0.1
14.6	6.38		401	2558	
15.0	1.69		401	678	
15.2	0.47		401	190	
15.5	1.51		401	606	
15.6	1.44		401	577	
15.8	0.88		401	353	
15.9	0.44		401	178	
16.1	0.63		401	253	
16.4	2.55		401	1021	
16.7	1.25		401	501	
17.4	7.25		401	2909	
17.7	1.64		401	656	
17.9	3.77		401	1512	
18.1	5.85	ΔIVs_{gal}	461	2695	
18.4	451.01	ΔIVs	461	207915	2.3
19.0	414.56	ΔIIa	503	208522	2.3
19.4	3.99		503	2005	
19.7	3.62		503	1819	
19.8	3.76		503	1891	
20.3	253.75	ΔIIIa	503	127637	1.4
21.0	1.23		533	657	
21.3	4.34		533	2312	
21.6	5.20		533	2773	
22.0	0.39		533	210	

(*) Area 232 nm x 2.5

Time (Min)	Area (265 nm)	Name	Molecular weight Mw (Da)	Area x Mw	% (w/w)
22.7	1.43		533	762	
22.8	1.61		533	858	
23.0	0.17		533	91	
23.2	25.09	GlcNS,3S	383	9610	0.1
23.5	112.64	GlcNS,6S	383	43142	0.5
24.2	7.97	UA(2,2,0)	581	4632	0.1
24.4	64.41	UA(2,2,0)	581	37424	0.4
24.7	0.20		533	108	
24.8	0.11		533	60	
25.2	4.58		533	2443	
25.5	4.06		533	2162	
25.8	30.66	ΔIIs_{gal}	563	17260	0.2
26.2	1372.31	ΔIIs	563	772609	8.6
27.2	2.39		563	1346	
27.4	11.40		563	6416	
27.8	959.88	◎IIIs	563	540412	6.0
28.6	8.95		583	5218	
29.2	4.69		583	2736	
29.6	202.98	ΔIa	605	122805	1.4
29.9	3.69		635	2345	
30.2	1.12		635	713	
30.5	6.10		635	3874	
31.1	0.27		635	174	
31.5	2.26		635	1435	
31.7	0.84		635	533	
31.9	0.26		635	166	
32.2	122.41	$\underline{\text{IIs}_{glu}}$	683	83605	0.9
32.9	78.21	$\Delta\text{IIa}\underline{\text{IVs}}$	1066	83374	0.9
33.3	87.87	Is_{id}	683	60014	0.7
33.9	16.31	$\Delta\underline{\text{IIs}}$	665	10844	0.1
34.0	63.33	GlcNS,6S,3S	485	30715	0.3
34.5	7.7	$\Delta\underline{\text{IIIs}}$	665	5131	0.1
35.2	1.4		635	887	
35.8	7.0		635	4436	
36.0	14.8	G(3,4,0)	946	14033	0.2
36.6	3.4		635	2177	
37.0	8.2		635	5230	
37.4	8344.6	ΔIs	665	5549169	61.8
39.3	94.1		665	62551	0.7
40.2	287.4	$\Delta\text{IIa}\underline{\text{IIs}}$	1168	335721	3.7
40.9	4.5		1230	5532	
41.1	5.5		1230	6715	

Time (Min)	Area (265 nm)	Name	Molecular weight Mw (Da)	Area x Mw	% (w/w)
41.4	6.4		1230	7865	
41.7	1.8		1230	2234	
42.7	0.3		1230	377	
43.2	3.2		1230	3932	
43.9	5.1		1230	6313	
44.4	19.2	G(3,5,0)	1048	20112	0.2
44.9	40.1	ΔIs	767	30729	0.3
46.1	2.8		1230	3443	
46.4	0.3		1230	346	
46.9	34.2	ΔIIsIIs	1228	42012	0.5
47.3	2.4		1230	2949	
47.6	2.2		1230	2742	
48.1	15.6	ΔIaIIs	1270	19791	0.2
48.4	1.6		1230	2028	
49.5	2.3		1230	2856	
51.0	3.3		1230	4029	
52.0	2.1		1230	2534	
53.2	1.6		1230	2003	
54.3	23.7	ΔIsIIs	1330	31539	0.4
55.8	3.7		1230	4562	0.1
Total				8974871.81	98.3

Table S9: Integration of peak areas determined from Figure S19 and integration results.

Building blocks analysis with the classical method

The quantification results by the classical method [7] of the heparins analyzed in Table 1 are gathered in table 10S. ΔGlyser$_{ox1}$ and ΔGlyser$_{ox2}$ correspond to the 2 oxidized glycoserines described in [7]. ΔGlyser correspond to the various glycoserines eluted before ΔIVa in the classical method, that is, not only the endogeneous glycoserine ΔGlcA-Gal-Gal-Xyl-Ser.

	Heparin	PMH	OMH	BMH
Unsaturated Building Blocks	ΔGlyser	0.8	0.5	0.6
	ΔIVa	2.2	1.8	3.2
	ΔGlyser$_{ox1}$	1.2	0.7	0.9
	ΔGlyser$_{ox2}$		0.4	0.4
	ΔIVs$_{gal}$	0.0	0.0	0.2
	ΔIVs	2.2	1.1	2.9
	ΔIIa	2.2	1.3	0.6
	ΔIIIa	1.3	0.5	1.1
	ΔIIs$_{gal}$	0.2	0.1	0.2
	ΔIIs	8.6	9.4	7.1
	ΔIIIs	6.0	5.9	25.5
	ΔIa	1.3	0.7	0.2
	ΔIIa-IVs	0.9	0.5	1.0
	ΔIIs	0.1		0.4
	ΔIIIs			1.2
	ΔIs	65.2	68.2	45.1
	ΔIIa-IIs	4.1	3.8	0.8
	ΔIs	0.4	0.4	1.7
	ΔIIs-IIs	0.3	0.9	0.5
	ΔIa-IIs	0.3	0.4	0.1
	ΔIs-IIs	0.4	1.5	1.0
Heparin	Sulfates/Carboxylates	2.44	2.52	2.26
	NAc	11.9	8.4	8.1
	6-OH	15.2	11.7	39.2
	2-OH	24.9	23.2	20.4
	3-OS	3.5	4.1	4.2

Table S10: Quantification of building blocks (% w/w) for PMH, OMH and BMH from Table 1 by the classical method [7] (NAc: % N-acetylated glucosamines; 6-OH: % 6-OH glucosamines; 2-OH: % 2-OH uronic acids; 3-OS: % 3-Osulfated glucosamines)

NMR comparison of heparins

The selective presence of NRE glucuronic acids in PMH is confirmed on NMR ^{1}H spectra (Figure S23).

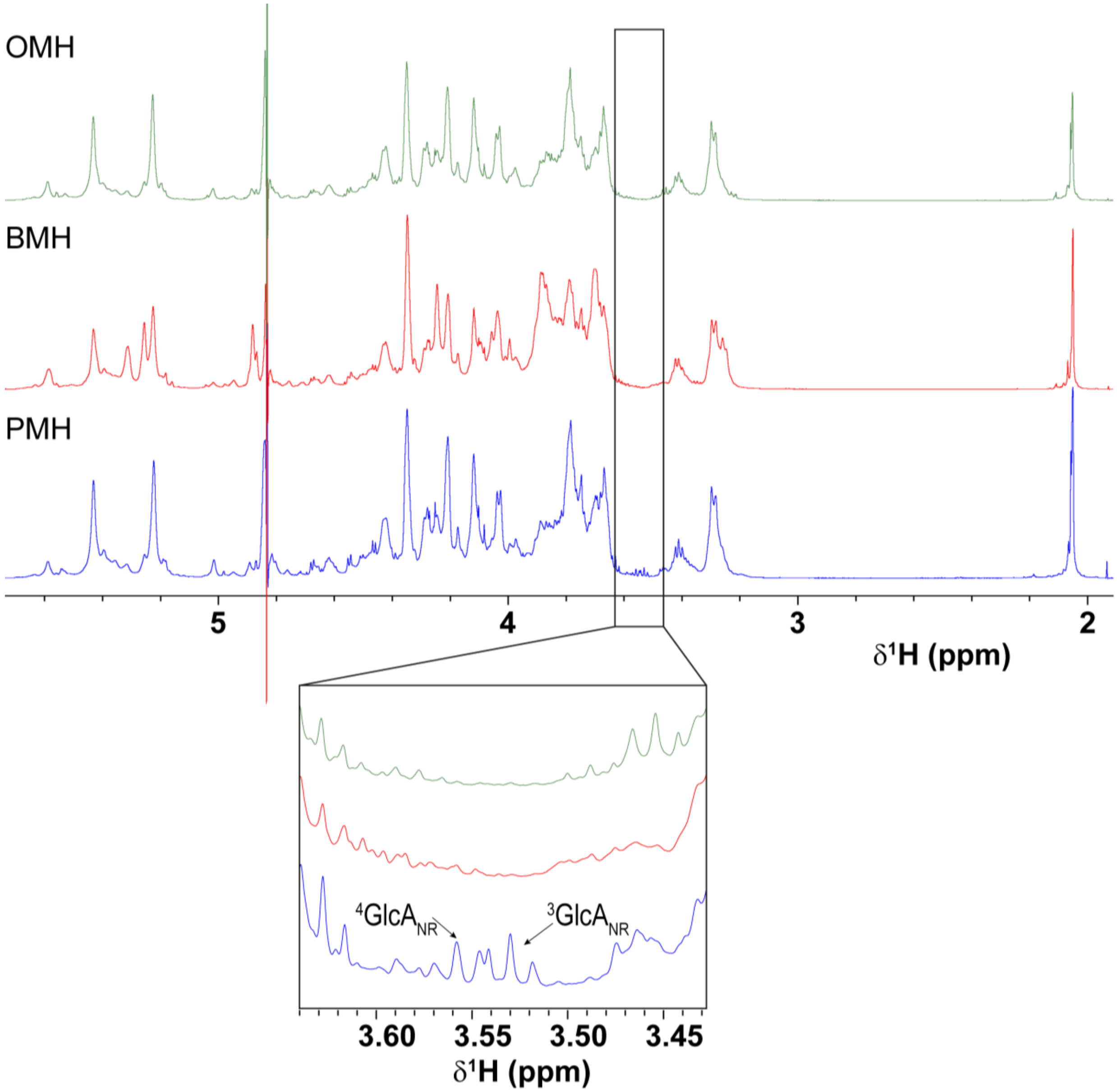

Figure S24: ^{1}H spectra of porcine, bovine and ovine mucosa heparins (D2O, 25°C, 800 MHz)
